# The genetic risk factor *CEL-HYB1* causes proteotoxicity and chronic pancreatitis in mice

**DOI:** 10.1016/j.pan.2022.11.003

**Published:** 2022-11-09

**Authors:** Karianne Fjeld, Anny Gravdal, Ranveig S. Brekke, Jahedul Alam, Steven J. Wilhelm, Khadija El Jellas, Helene N. Pettersen, Jianguo Lin, Marie H. Solheim, Solrun J. Steine, Bente B. Johansson, Pål R. Njølstad, Caroline S. Verbeke, Xunjun Xiao, Mark E. Lowe, Anders Molven

**Affiliations:** a The Gade Laboratory for Pathology, Department of Clinical Medicine, University of Bergen, Bergen, Norway; b Center for Diabetes Research, Department of Clinical Science, University of Bergen, Norway; c Department of Medical Genetics, Haukeland University Hospital, Bergen, Norway; d Department of Pediatrics, Washington University School of Medicine, St. Louis, MO, USA; e Pediatric and Youth Clinic, Haukeland University Hospital, Bergen, Norway; f Department of Pathology, Oslo University Hospital Rikshospitalet, Oslo, Norway; g Department of Pathology, Institute of Clinical Medicine, University of Oslo, Oslo, Norway; h Department of Pathology, Haukeland University Hospital, Bergen, Norway; i Section for Cancer Genomics, Haukeland University Hospital, Bergen, Norway

**Keywords:** Chronic pancreatitis, Carboxyl ester lipase, Genetic risk factor, Knock-in mouse model, Variable number of tandem repeats

## Abstract

**Background & aims:**

The *CEL* gene encodes the digestive enzyme carboxyl ester lipase. *CEL-HYB1*, a hybrid allele of *CEL* and its adjacent pseudogene *CELP*, is a genetic variant suggested to increase the risk of chronic pancreatitis (CP). Our aim was to develop a mouse model for *CEL-HYB1* that enables studies of pancreatic disease mechanisms.

**Methods:**

We established a knock-in mouse strain where the variable number of tandem repeat (VNTR) region of the endogenous mouse *Cel* gene was substituted with the mutated VNTR of the human *CEL-HYB1* allele. Heterozygous and homozygous *Cel-HYB1* mice and littermate wildtype controls were characterized with respect to pancreatic pathology and function.

**Results:**

We successfully constructed a mouse model with pancreatic expression of a humanized CEL-HYB1 protein. The *Cel-HYB1* mice spontaneously developed features of CP including inflammation, acinar atrophy and fatty replacement, and the phenotype became more pronounced as the animals aged. Moreover, *Cel-HYB1* mice were normoglycemic at age 6 months, whereas at 12 months they exhibited impaired glucose tolerance. Immunostaining of pancreatic tissue indicated the formation of CEL protein aggregates, and electron microscopy showed dilated endoplasmic reticulum. Upregulation of the stress marker BiP/GRP78 was seen in pancreatic parenchyma obtained both from *Cel-HYB1* animals and from a human *CEL-HYB1* carrier.

**Conclusions:**

We have developed a new mouse model for CP that confirms the pathogenicity of the human *CEL-HYB1* variant. Our findings place *CEL-HYB1* in the group of genes that increase CP risk through protein misfolding-dependent pathways.

## 1. Introduction

Chronic pancreatitis (CP) is a complex and progressive inflammatory disease. The risk of developing CP is known to be influenced by life-style factors, such as alcohol and smoking, and by multiple genetic loci [1]. The genetic risk variants can be classified into mechanistic pathways that describe their pathogenic effects. The largest group of such variants belong to the trypsin-dependent pathway, covering *PRSS1*, *SPINK1*, *CTRC* and other genes that determine the degree of intrapancreatic trypsin activation [2,3]. Another class consists of variants of genes involved in ductal secretion processes, exemplified by *CFTR* and *TRPV6* [4]. Moreover, some patients carry rare variants of digestive enzyme genes that induce proteotoxic endoplasmic reticulum (ER) stress within the pancreatic acinar cells. The latter group constitutes the protein misfolding-dependent pathway of genetic susceptibility variants in CP [5]. Risk variants of the *CPA1* gene and some *PRSS1* variants can be assigned to this group [6,7].

A third gene suggested to be a member of the protein misfolding-dependent pathway is *CEL*, which encodes carboxyl ester lipase. The CEL enzyme, also known as bile salt-dependent or bile salt-stimulated lipase, hydrolyzes dietary fat, cholesteryl esters and fat-soluble vitamins in the intestine [8]. CEL is comprised of a globular protein domain that carries out the enzyme's catalytic function and an intrinsically disordered tail region containing a repeated motif of 11 amino acids. The tail is encoded by a variable number of tandem repeat (VNTR) sequence present in exon 11 of the *CEL* gene [8]. Deletions of one base pair within the VNTR change the reading frame and lead to a new tail region with altered biochemical properties [9-11]. Such *CEL* mutations are very rare and can cause the inherited disease MODY8, clinically characterized by diabetes, pancreatic exocrine dysfunction and a variety of pathological changes in the pancreatic parenchyma [12,13]. Notably, some of the deletion carriers are diagnosed with CP [13]. The CEL protein variants arising because of the single-base pair deletions have a propensity to aggregate and to upregulate ER stress markers and are, therefore, considered to be proteotoxic [11,14].

In 2015, we reported a novel type of pathogenic *CEL* variant [15]. *CEL* is localized on chromosome 9 in tandem with a pseudogene copy (*CELP*) of itself, and the new variant had probably arisen due to non-allelic, homologous recombination within the *CEL* locus. In this way, the first ten exons of *CEL* became fused to the last exon of the pseudogene, resulting in a hybrid allele between *CEL* and *CELP*. Hence, the allele was named *CEL-HYB1*. Because the VNTR region of *CEL-HYB1* originates from *CELP*, it is predicted to encode a protein tail with only three copies of the repeated amino acid motif.

*CEL-HYB1* was found to be five-fold more frequent in three European cohorts of idiopathic CP than in healthy controls [15]. This variant has also been observed in two Norwegian families, where the probands had additional risk factors (smoking, pancreas divisum) for developing pancreatitis [16]. However, a replication effort in Polish pediatric patients with CP found only a two-fold, non-significant overrepresentation of *CEL-HYB1* [17]. The allele has also been investigated in pancreatic cancer patients with negative results regarding disease association [18,19]. Notably, *CEL-HYB1* was absent from three different Asian cohorts of CP patients [20]. Instead, an alternative hybrid allele (*CEL-HYB2*), with a premature stop codon in exon 10, was discovered. *CEL-HYB2* had a combined carrier frequency of 1.7% among both the pancreatitis cases and the controls, and therefore showed no disease association [20,21].

Thus, whereas the *CEL* deletion variants in MODY8 have a strong pathogenic effect, leading to a highly penetrant, Mendelian disease, the role of *CEL-HYB1* in exocrine pancreatic disease is somewhat unclear. We therefore sought to construct an animal model that could elucidate the effects of *CEL-HYB1* in the pancreas. To this end, we have established a mouse strain where the endogenous VNTR of the mouse *Cel* gene has been substituted with the VNTR of the human *CEL-HYB1* allele by homologous recombination. Herein, we report the pancreatic phenotype of this model, demonstrating that it leads to morphological changes in the pancreas consistent with CP and that protein misfolding and proteotoxicity are likely to be part of the disease mechanism.

## 2. Methods

### 2.1. Sequences and nomenclature

Accession number for the *Mus musculus* strain C57BL/6N *Cel* gene is MGP_C57BL6NJ_G0025943 (Mouse Genome Informatics database, www.informatics.jax.org). Accession number for the human *CEL* gene is ENSG00000170835 (EMBL-EBI Ensembl genome browser, www.ensembl.org). We will use mCEL when referring to the protein encoded by the mouse gene and CEL as a reference to human CEL or the mammalian protein in general. The protein expressed by the human *CEL-HYB1* allele will be denoted CEL-HYB1, whereas the “humanized” mouse protein, expressed in the knock-in mouse, will be named mCEL-HYB1.

### 2.2. Expression of CEL in HEK293T cells and lipase activity measurement

A cDNA clone encoding the full-length mCEL protein (Clone ID: OMu05057C; NM_009885.2) was purchased from Genscript and sub-cloned into the plasmid pcDNA3.1 (Addgene) by using restriction sites *Hin*dIII/*Bam*HI. The pcDNA3.1/*Cel* construct was then used to express the normal mCEL protein. To express the humanized mCEL-HYB1 protein, a 246 bp *Kpn*I/*Not*I DNA fragment corresponding to the VNTR of human *CEL-HYB1* was synthesized by Genscript to replace the mouse VNTR region of the pcDNA3.1/*Cel* plasmid. Notably, the resulting pcDNA3.1/*Cel-HYB1* plasmid was constructed without any of the *CEL-HYB1* SNPs described by Cassidy et al. [22]. Both pcDNA3.1/*Cel* and pcDNA3.1/*Cel-HYB1* were confirmed by Sanger sequencing before being transiently transfected into HEK293T cells. Forty-eight h after transfection, cell medium was removed and exchanged with 1.2 ml Reduced-Serum Opti-MEM medium (Gibco). Twenty h post-medium exchange, conditioned medium, soluble cell lysate and the insoluble pellet were harvested and analyzed for CEL expression. The conditioned medium was also used for measuring lipase activity. Protocols for cell transfection, cell fractionation, western blotting and lipase activity measurements have previously been described in detail by Cassidy et al. [22].

### 2.3. Animal study protocol approval

The Laboratory Animal Facility, Faculty of Medicine, University of Bergen, Norway was used for the housing and care of the majority of animals. The Animal Care and Use Programs at University of Bergen are accredited by the Association for Assessment and Accreditation of Laboratory Animal Care International. Experiments were approved by the Norwegian Animal Research Authority with the approval IDs FOTS 13902 (breeding) and FOTS 13510 (experiments). The experiments were conducted according to the European Convention for the Protection of Vertebrates Used for Scientific Purposes. A small number of animals (for immunoblotting, immunostaining, and electron microscopy) were housed and studied at Washington University in St. Louis in accordance with the policies and guidelines set forth by the Institutional Animal Care and Use Committee (IACUC; protocol ID: 19–1109).

### 2.4. Generation of the Cel-HYB1 mouse

Construction of the targeting vector and generation of the *Cel-HYB1* mouse strain were done by genOway (Lyon, France). The strain was established on a C57BL/6N genetic background (Charles River Laboratories, Lyon, France). The mouse *Cel* gene is located on chromosome 2, spans around 7.5 kb and contains 11 exons. The targeting vector for homologous recombination consisted of a short arm (*Cel* exon 8–10 region), a middle arm (exon 11 coding sequence) and a long arm (exon 11 downstream sequence). A neomycin resistance cassette (positive selection marker) flanked by loxP sites was inserted in intron 10, and a diphtheria toxin negative selection marker was present outside the homology arms. In the middle arm, the three VNTR segments of the endogenous mouse *Cel* gene were replaced by a synthetic DNA fragment corresponding to the three VNTR segments of the human *CEL-HYB1* allele [15]. The final targeting vector was validated by DNA sequencing.

The targeting vector was electroporated into C57BL/6N embryonic stem (ES) cells. G418-resistant clones were harvested and screened by PCR and Southern blot analysis to confirm the homologous recombination event on both sides of *Cel* exon 11. Correctly recombined ES cell clones were injected into blastocysts and implanted into pseudopregnant females. Male chimeric progeny (chimerism rate >50%) were bred with female C57BL/6N Cre deleter mice to remove the neomycin resistance cassette. Resultant animals were DNA-sequenced to verify the integrity of the targeted region plus a minimum of 1 kb upstream and downstream of both homology arms. The sequence of the critical exon 11 region is given in Supplementary Fig. 1. Verified animals were then transferred to the University of Bergen and used to establish a colony of *Cel-HYB1* mice by backcrossing with C57BL/6N wild type mice (Charles River Laboratories, Lyon, France). Some verified *Cel-HYB1* mice were further transferred from University of Bergen to Washington University in St. Louis, where they were maintained and expanded on the same C57BL/6N genetic background.

### 2.5. Animal maintenance

*Cel-HYB1* mice were kept on the C57BL/6N background and housed on a 12-h light/dark cycle with *ad libitum* access to water and food. Both male and female animals were studied. The number of animals, age and genotype used in each experiment are shown in figures. All control animals were wildtype littermates. At the age indicated, the mice were euthanized, and tissue samples collected. For both pancreas, white and brown adipose tissue, the weight was measured and presented as percent of body weight. The adipose tissues and half of the pancreas were fixed in formalin for histology. The other half of the pancreas was snap-frozen in liquid nitrogen and stored at −80 °C until protein extraction and trypsin analysis.

### 2.6. Genotyping

For genotyping of *Cel-HYB1* mice, we employed primers that flank the remaining LoxP site in intron 10 of the knock-in allele. Sequences were 5′-GCA AAC TTC TTA TTT ATC CTC AAG CC TTG G-3’ (forward primer) and 5′-GTT ATC GTC TTA GTG ATG TCC AGG TAG TTG C-3’ (reverse primer). Amplicon sizes from the wild-type and knock-in alleles were 303 and 394 bp, respectively.

### 2.7. Western blotting and pancreas cell fractionation

Mouse pancreatic tissue was homogenized with a pestle in ice-cold Trident RIPA lysis buffer (Gene Tex, GTX400005) supplemented with cOmplete Protease Inhibitor Cocktail (Roche). The homogenate was incubated on a rotary wheel for 20 min at 4 °C and centrifuged for 15 min at 13 000×*g* rpm and 4 °C. The supernatant was isolated and 10 μg protein analyzed by sodium dodecyl sulfate polyacrylamide gel electrophoresis (SDS-PAGE; 10%), followed by transfer to a polyvinylidene difluoride membrane by semi-wet blotting using XCell Blot Module chambers (Invitrogen). After blocking with 5% non-fat milk in PBS supplemented with 0.05% Tween 20 for 1 h, the membrane was incubated with primary antibody overnight at 4 °C, followed by secondary antibody for 1 h at room temperature. The bands were detected using ECL Plus Western Blotting Substrate (Pierce) and the ChemiDoc MP Imaging System (Bio-Rad). Antibodies and dilutions were as follows: rabbit anti-CEL (against the truncated human CEL variant pV562Δ, see ref. 14), 1:5000; mouse anti-β-actin (Sigma, A5441), 1:1000; HRP-conjugated donkey anti-mouse (Santa Cruz, sc-2306), 1:5000; HRP-conjugated goat anti-rabbit (ThermoFisher, 65–6120), 1:5000. The rabbit antibody against part of the VNTR-encoded CEL-HYB1 protein sequence (DRQLRVCPRP) was custom-made (Davids Biotechnologie, Regensburg, Germany) and employed at dilution 1:300.

For cell fractionation, 20–30 mg mouse pancreas tissue was homogenized by sonication (5 × 10 s on ice) in RIPA buffer (Sigma, 20–188) with protease and phosphatase inhibitor cocktails, at a ratio of 30 μl RIPA/1 mg tissue. After homogenization, whole-cell lysate, detergent-soluble and insoluble fractions were processed, separated by SDS-PAGE, and immunoblotted for CEL as described previously [22]. Rat monoclonal anti-α-tubulin antibody (Santa Cruz, sc-53029; 1:2000) was used for endogenous control. The band density sum was used to calculate the percentage of the amount of insoluble CEL relative to that of total CEL protein.

### 2.8. Histology and evaluation of pathology

Formalin-fixed (10%) and paraffin-embedded (FFPE) pancreatic tissue was sectioned (4–5 μm) and stained with hematoxylin and eosin (HE) using a standard protocol. For Masson's trichrome staining, the Ventana Trichrome staining kit (Roche, 860-031) was used. For evaluation of morphological changes, HE sections were blindly (i.e. without access to genotype, age or sex) evaluated by an expert in pancreas pathology (C.S.V.). Inflammation, acinar atrophy and fatty replacement were assessed, and a score of normal morphology (0), mild to moderate changes (1), extensive changes (2) were given (Supplementary Table 1).

### 2.9. Immunohistochemistry

FFPE pancreatic tissue sections (4–5 μm) were dried overnight at 56 °C, deparaffinized in xylene, gradually rehydrated with ethanol, and washed with distilled water and PBS containing Tween 0.05%. Staining for CEL was then carried out as previously described [23] using anti-CEL antibody (Sigma-Aldrich, HPA052701; 1:200). For the other markers, the staining was performed on a Bond Rx autostainer (Leica Biosystems) using standard protocols. The sliced FFPE pancreatic sections were first enzymatically pre-treated for 20 min to expose epitopes by using the Bond Enzyme Pre-Treatment Kit (Leica, AR9551, 1:1000 dilution). Primary antibodies for other markers were rat monoclonal anti-F4/80 antibody (Thermo Fisher Scientific, #14480182; 1:200), rat monoclonal anti-B220/CD45R antibody (Novus Biologicals, #10077420; 1:10000), rabbit polyclonal anti-CD45 antibody (Abcam, ab10558; 1:2000). Bond Polymer Refine Detection (Leica Biosystems) was used according to the manufacturer's protocol with appropriate secondary antibodies. After staining, sections were dehydrated and cover-slipped, and whole-slide scanning (40x) was performed on an Aperio AT2 (Leica Biosystems). The slides were analyzed using Aperio ImageScope software (Leica Biosystems).

### 2.10. Immunofluorescence

After deparaffinization, the FFPE pancreatic slides were incubated in Tris-EDTA buffer, pH 9 (CEL staining) or citric acid-based buffer, pH 6 (BiP staining) in a pressurized heating chamber. Slides were then incubated with goat serum blocking solution and incubated at 4 °C overnight with antibodies against CEL (Sigma-Aldrich, HPA052701; 1:100) or BiP (Abcam, ab21685 1:400). After washing with PBS-Tween, slides were incubated with goat anti-rabbit secondary antibody Alexa Fluor 594 (Invitrogen, A-11012; 1:1000) for 1 h at room temperature. Slides were mounted with ProLong Gold Antifade Mountant (Invitrogen) after DAPI staining. Imaging was performed by using a SP8 AOBS confocal microscope (Leica Microsystems). The obtained images were merged and processed using Photoshop CC and Adobe Illustrator CC (Adobe Systems) with BiP staining changed to green by the software for illustrative purposes. Focal areas of positive staining were selected for imaging.

### 2.11. Transmission electron microscopy

Mice pancreatic tissue was fixed in 2.5% glutaraldehyde in 0.1 M sodium cacodylate buffer for 24 h at 4 °C. Post-fixation was performed for 1 h on ice in 1% osmium tetroxide (Electron Microscopy Sciences, #19134) diluted in 0.1 M sodium cacodylate buffer, followed by two washing steps with ultrapure water. The samples were dehydrated using a graded ethanol series (30–100%) before being incubated for 15 min in a 1:1 solution of 100% ethanol:propylene oxide. Samples were transferred to 100% propylene oxide (15 min) before gradually introducing agar 100 resin (Agar Scientific, R1031). Samples were then transferred to a small drop of 100% resin and excess propylene oxide was allowed to evaporate before a new transfer to 100% resin. The samples were placed in molds, left at room temperature overnight, and then incubated at 60 °C for 48 h to polymerize. Finally, 70 nm thin sections were cut from the resin block and post-stained with uranyl acetate and Reynold's lead. Cells with pathology were arbitrarily selected for imaging which were acquired by a JEM-1400 Plus transmission electron microscope (JEOL Inc.) operating at 120 KeV.

### 2.12. Serum amylase activity

Blood was collected through post-mortem cardiac puncture, transferred to EDTA-coated tubes and left at room temperature for 20 min. After centrifugation (3000×*g*, 10 min, 4 °C), serum was pipetted off for amylase determination using 2-chloro-ρ-nitrophenyl-α-D-maltotrioside (Pointe Scientific, A7564-120) as substrate. Serum (4 μl) was diluted with 6 μl 0.9% NaCl and mixed with 190 μl substrate to start the reaction. The increase in absorbance due to release of 2-chloro-notrophenol was followed for 2 min in a plate reader at 405 nm. Amylase activity (rate of substrate cleavage) was expressed as mOD/min.

### 2.13. Intrapancreatic trypsin activity

Frozen pancreatic tissue samples were homogenized in MOPS buffer (250 mM sucrose, 5 mM MOPS (pH 6.5), 1 mM MgSO4) using 30 μl buffer per 1 mg tissue. Homogenization was performed manually on liquid nitrogen with a pestle, followed by sonication (5 × 10 s, 132 kHz) on ice and centrifugation for 3 min (1500×*g*, 4 ° C). The supernatant was dissolved in assay buffer (0.1 M Tris-HCl (pH 8), 1 mM CaCl_2_, 0.05% Tween) to a total volume of 50 μl, using a supernatant volume that corresponded to 10–30 μg total protein. Trypsin activity in the supernatant was then determined by adding 150 μl of 200 μM fluorescent substrate (Boc-Gln-Ala-Arg-AMC·HCl; Bachem, USA) dissolved in assay buffer to initiate the reaction. Increase in fluorescence was followed for 5 min in a plate reader at wavelengths 380 nm (excitation) and 460 nm (emission). Trypsin activity (rate of substrate cleavage) was expressed as relative fluorescent units (RFU) per sec and normalized to total protein content in the analyzed sample.

### 2.14. RNA isolation, reverse transcription and quantitative real-time PCR (qPCR)

Total RNA was extracted from mouse pancreas (10–20 mg) using the RNeasy Plus Mini Kit (Qiagen). For reverse transcription, RNA (1 μg) and the Quantiscript Reverse Transcription Kit (Qiagen) was used to synthesize complementary DNA. TaqMan gene expression assays and the TaqMan Fast Advanced Master Mix (Applied Biosystems) were used to determine the mRNA expression levels for mouse *Hspa5* and *Ddit3* (Assay ID Mm00517691_m1 and Mm01135937_g1, respectively). The mouse *Gapdh* assay (ID Mm99999915_g1) was used as reference. Expression was calculated using the ΔΔC_T_ method, and the results were presented as fold change calculated with the formula 2^−ΔΔCT^.

### 2.15. Glucose homeostasis

Glucose was measured from blood collected from the tail vein using a FreeStyle glucometer (Abbott). For random glucose measurements, mice were tested in the morning after *ad libitum* access to water and food. For glucose tolerance testing (GTT), the animals were fasted overnight (16 h) and injected intraperitoneally with glucose at a dose of 2 g/kg body weight. Blood glucose concentrations were measured at 0, 15, 30, 60, 90 and 120 min. Area under the curve (AUC) was calculated using GraphPad Prism version 9.2.0.

### 2.16. Human samples

Human pancreatic tissue samples were obtained from a biobank of pancreatic neoplastic lesions [23,24]. The patients had consented to the study, which was approved by the Regional Ethical Committee of Western Norway (REK Vest 2013/1772) and performed according to the Helsinki Declaration.

### 2.17. Statistics

Results were plotted as individual data points, with the mean and standard deviation (SD) or standard deviation of the mean (SEM) as indicated. Differences of means between two groups were analyzed by two-tailed unpaired *t*-test. P < 0.05 was considered statistically significant.

## 3. Results

### 3.1. Comparison of the humanized mCEL-HYB1 protein with normal mCEL

A *Cel-HYB1* plasmid was made by replacing the mouse *Cel* VNTR with the human VNTR from the *CEL-HYB1* allele. This construct was expressed in HEK293T cells and compared with a construct encoding mCEL, the normal mouse CEL protein (Supplementary Fig. 2A). The mCEL-HYB1 protein was less secreted into the medium than mCEL (Supplementary Fig. 2B). Moreover, the abundance of mCEL-HYB1 was lower in the soluble cell lysate and higher in the insoluble pellet compared with mCEL (Supplementary Fig. 2B). We also measured the enzyme activity in the conditioned media, finding that mCEL-HYB1 exhibited around 50% reduced lipase activity compared to the normal mouse enzyme (Supplementary Fig. 2C). Thus, by introducing the *CEL-HYB1* VNTR, the properties of mCEL changed in the same direction as previously described for the human CEL-HYB1 protein variant [15,16,22].

### 3.2. Generation of the Cel-HYB1 knock-in mouse

**Fig. 1.**
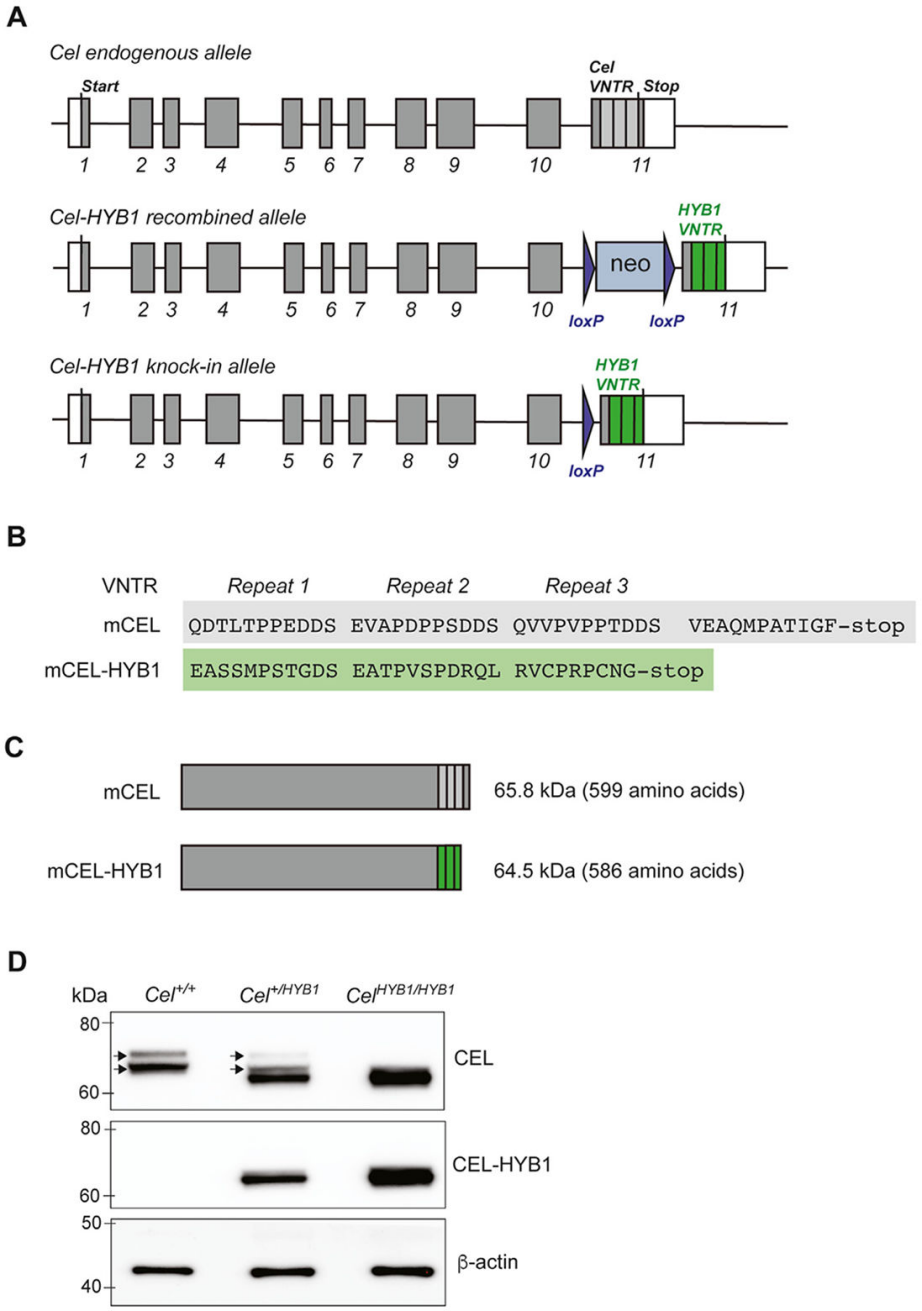
Generation of a humanized *Cel-HYB1* knock-in mouse strain. (A) Homologous recombination in embryonic stem cells was used as targeting strategy to create animals carrying the human *CEL-HYB1* VNTR region in mouse *Cel* exon 11. As positive selection marker, a neomycin resistance cassette (neo) flanked by loxP sites was introduced in intron 10. This cassette was subsequently removed by breeding with a Cre deleter strain. The numbered boxes illustrate the eleven exons of *Cel* with white rectangles representing noncoding regions. In exon 11, the three repeated segments in the mouse *Cel* VNTR (light grey) and in the VNTR of human *CEL-HYB1* (green) are indicated. Elements of the figure are not drawn to scale. (B) C-terminal amino acid sequences of the normal mouse Cel protein (mCEL) and the humanized mCEL-HYB1 protein variant. (C) Schematic comparison of the mCEL and mCEL-HYB1 proteins. (D) Expression of mCEL protein and mCEL-HYB1 in 12-week-old male mice. Western blot was performed on pancreas lysates from wildtype littermate controls (*Cel*^*+/+*^), heterozygous (*Cel*^*+/HYB1*^) and homozygous (*Cel*^*HYB1/HYB1*^) mice. The antibody either recognized the CEL globular domain (upper panel) or was specific for the tail of the CEL-HYB1 variant (middle panel). Anti β-actin was visualized for loading control. One representative lysate of each genotype is shown. The black arrows indicate the two bands observed for normal mCEL.

We then set out to create a *Cel-HYB1* mouse via homologous recombination in ES cells as described in Methods. The model is a knock-in strain, in which the normal VNTR of *Cel* exon 11 has been replaced by the VNTR of the human *CEL-HYB1* allele (Fig. 1A). The mouse *Cel* VNTR consists of three segments encoding 11 amino acids each, followed by a tail of 11 unrelated amino acids. The amino acid sequence encoded by the *Cel-HYB1* VNTR is altered compared with the VNTR of the mouse *Cel* gene. In addition, the third segment of the *Cel-HYB1* VNTR encodes only nine amino acids, and there is no additional tail sequence (Fig. IB). This implies that the humanized protein of the *Cel-HYB1* strain is slightly shorter than the normal mCEL protein (Fig. 1C).

The founder mice of the *Cel-HYB1* strain were DNA-sequenced to verify that *Cel* exon 11 contained the intended change and that no other genetic events had taken place in the target region during creation of the strain (Supplementary Fig. 1). To verify that the mCEL-HYB1 protein was expressed as desired, pancreatic protein extracts were made from wildtype (*Cel*^*+/+*^), heterozygous (*Cel*^*+/HYB1*^) and homozygous (*Cel*^*HYB1/HYB1*^) animals. For *Cel*^*+/+*^ mice, a double band of the expected size was observed (Fig. 1D), probably representing two differentially glycosylated forms of normal mCEL, as observed for the human CEL protein [11]. This double band was also present in heterozygous *Cel*^*+/HYB1*^ animals, although at a lower level, whereas it was not seen in the homozygous *Cel*^*HYB1/HYB1*^ knock-ins. As predicted, pancreatic extracts from mice carrying the *Cel-HYB1* allele exhibited an additional band of slightly lower molecular weight, not seen in wild type controls (Fig. 1D). We also employed a custom-made antibody produced against the tail region of the human CEL-HYB1 protein. No band was detectable in wildtype mice, whereas a band of the expected size was seen in the *Cel-HYB1* animals, with the strongest signal in homozygotes (Fig. 1D). The immunoblots were therefore consistent with pancreatic expression of the desired mCEL-HYB1 protein.

### 3.3. Growth and development of Cel-HYB1 mice

**Fig. 2.**
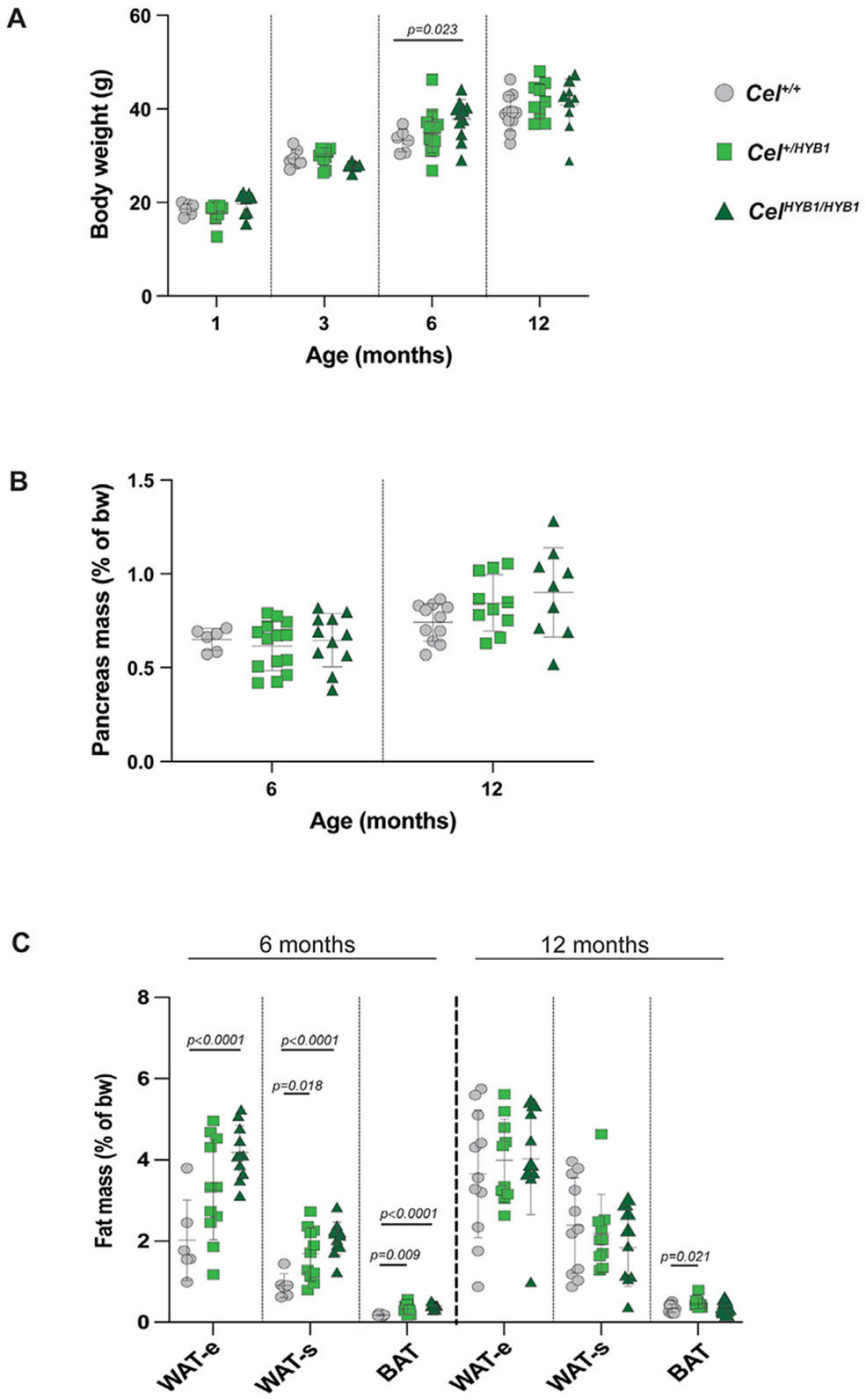
Body weight development and relative organ weights in male *Cel-HYB1* mice. Control (*Cel*^*+/+*^), heterozygous (*Cel*^*+/HYB1*^) and homozygous (*Cel*^*HYB1/HYB1*^) animals were compared. Individual values with mean (horizontal bar) ± SD are shown. *P*-values are listed for statistically significant differences between groups. (A) Body weight at age 1, 3, 6 and 12 months. (B, C) Pancreas and fat tissue mass of 6- and 12-month-old mice expressed as percent of body weight. Group sizes: n = 6–14. WAT, white adipose tissue; e, epididymal; s, subcutaneous; BAT, brown adipose tissue.

We monitored female and male *Cel-HYB1* mice for up to 6 and 12 months, respectively. Except for one homozygous male mouse that had to be sacrificed at age 8 months due to deteriorating general condition, heterozygous and homozygous animals showed no obvious physical or behavioral changes and bred normally. For homozygous *Cel-HYB1* male mice, we observed a significant increase in body weight compared to controls at age 6 months but not 12 months (Fig. 2A). Relative pancreatic mass was similar between the genotypes (Fig. 2B). At 6 months of age, a significant increase in the relative mass of both white (epididymal and subcutaneous) and brown adipose tissue was noted in mutant male mice compared to controls (Fig. 2C). At age 12 months, fat mass increase was statistically significant only for brown fat in heterozygotes.

At age 6 months, body weight was similar for female *Cel-HYB1* mice and controls (Supplementary Fig. 3A). At this age, we observed a significant decrease in relative pancreas weight for heterozygous females (Supplementary Fig. 3B). Moreover, there was generally a decreased relative mass of white, but not brown adipose tissue (Supplementary Fig. 3C). Thus, regarding body and tissue weight, there seemed to be different phenotypic effects of the *Cel-HYB1* allele in male and female mice at age 6 months. In particular, the effect on fat depots was opposite in the two sexes.

### 3.4. Cel-HYB1 mice develop chronic pancreatitis

**Fig. 3.**
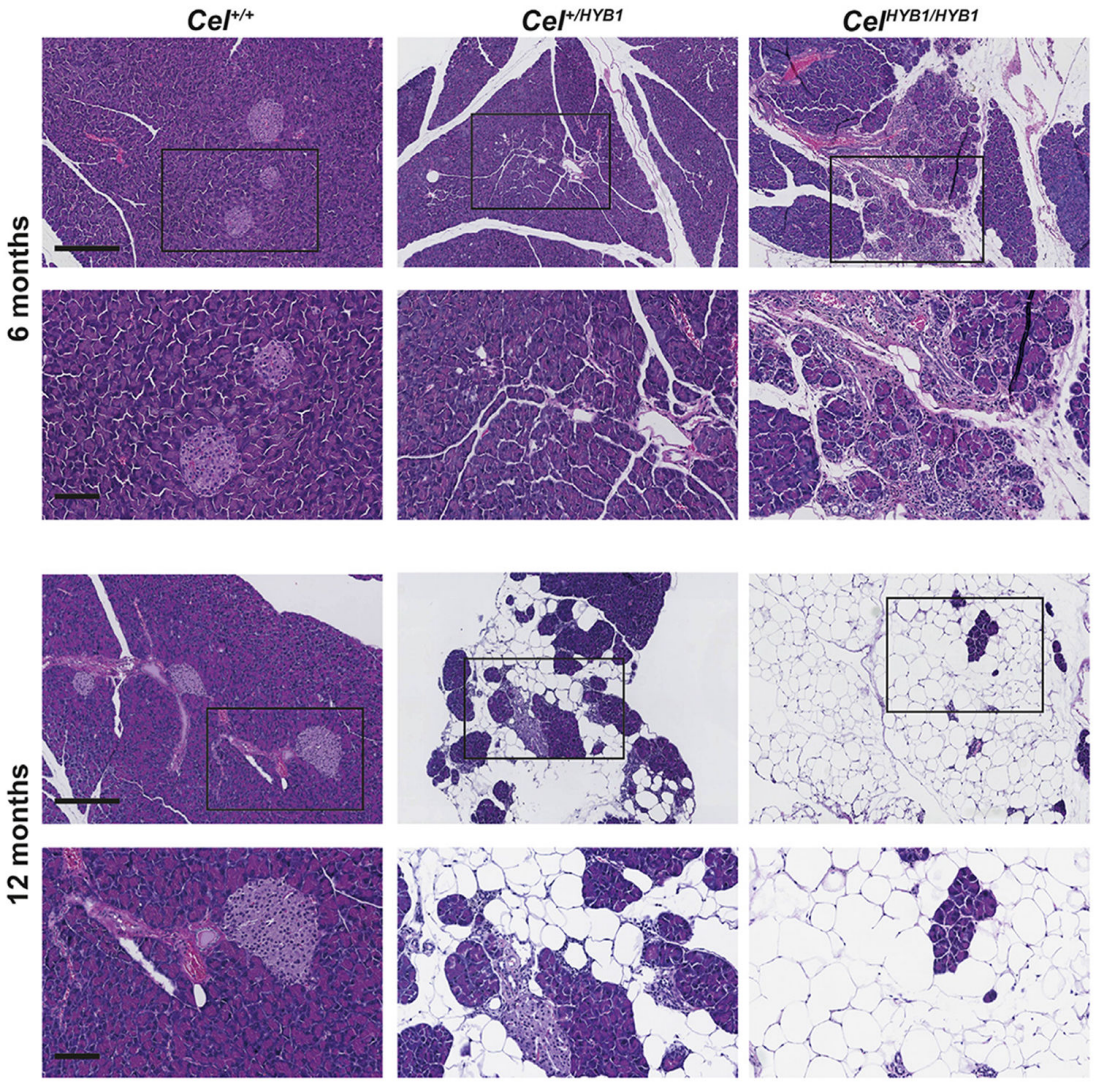
Pancreas histology in *Cel-HYB1* mice. Representative HE-stained pancreatic sections are shown from male controls (*Cel*^*+/+*^), heterozygous (*Cel*^*+/HYB1*^) and homozygous (*Cel*^*HYB1/HYB1*^) animals at age 6 and 12 months. Morphological changes typical for CP (inflammation, acinar atrophy, fatty replacement) are present, with more pronounced pathology with increasing age. Scale bars are 300 μm (for images with low magnifications) and 100 μm (high magnifications).

Based on examination of HE sections, we did histopathologic scoring of the pancreas from all mice included in this study (Supplementary Table 1). Male *Cel*^*+/HYB1*^ mice aged 6 months showed somewhat loosely packed acinar architecture and focal infiltration of inflammatory cells in three of 11 evaluated mice (Supplementary Table 1, Fig. 3). The early histological changes appeared patchy as the damage was restricted to some acinar lobules, while the surrounding exocrine tissue was normal. For the *Cel*^*HYB1/HYB1*^ littermates, more advanced pathological changes that also included acinar atrophy and fatty replacement were observed in six of 11 mice.

At age 12 months, the pancreata of all but one of 19 examined male *Cel*^*+/HYB1*^ or *Cel*^*HYB1/HYB1*^ mice showed widespread pathology, dominated by inflammation, fatty replacement and acinar cell loss (Fig. 3). In the atrophic areas of mice aged 12 months, the islets of Langerhans remained abundant, appeared somewhat enlarged and sometimes fused (Supplementary Fig. 4A). Overall, the histopathological changes were similar for hetero- and homozygous male mice (Supplementary Table 1). However, one homozygous animal had to be sacrificed at age 8 months. Here, the pancreas showed extensive fibrosis and inflammation, duct dilation as well as large areas with acinar-to-ductal metaplasia and loss of exocrine parenchyma (Supplementary Fig. 4B). Acinar-to-ductal metaplasia was never seen in 6-month *Cel-HYB1* mice but was a regular feature of animals aged 12 months.

For *Cel-HYB1* females at 6 months, we observed similar histological changes as in males (Supplementary Fig. 5A). Inflammation appeared to be more intense and present in 16/23 animals (Supplementary Table 1). Few animals were available for evaluation at 12 months, but the trend looked the same (Supplementary Table 1).

**Fig. 4.**
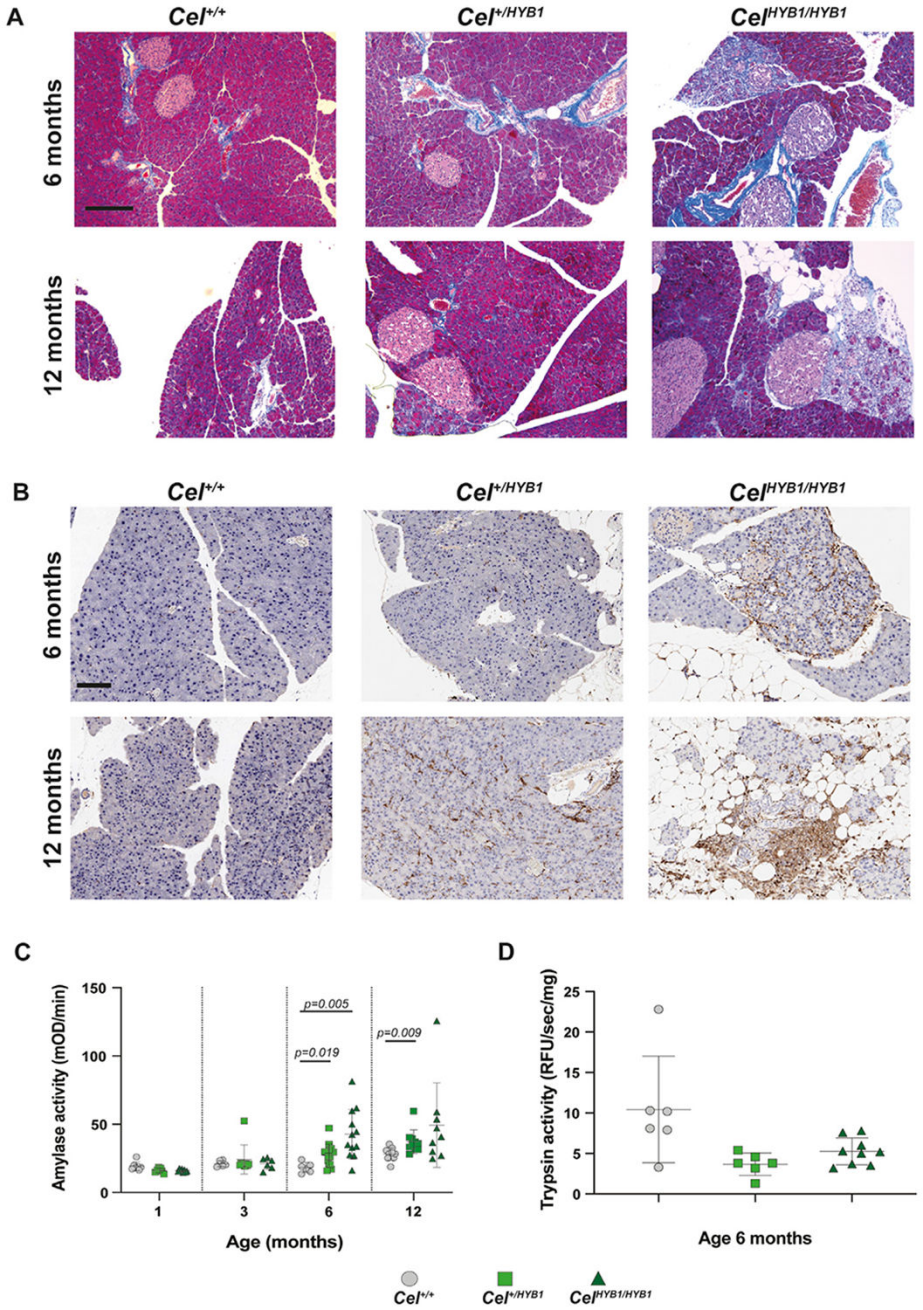
Fibrosis, inflammation and pancreatitis markers in *Cel-HYB1* mice. (A) Fibrosis demonstrated by Masson's trichrome staining and (B) immunostaining for the macrophage marker F4/80. Representative pancreatic sections are shown from male control (*Cel*^*+/+*^), heterozygous (*Cel*^*+/HYB1*^) and homozygous (*Cel*^*HYB1/HYB1*^) animals at age 6 and 12 months. Scale bars are 200 μm. (C) Serum amylase activity at ages 1, 3, 6 and 12 months, and (D) intrapancreatic trypsin activity at age 6 months in *Cel-HYB1* and control male mice. Individual values with mean (horizontal bar) ± SD are shown. When comparison between groups resulted in statistical significance, *p*-values are listed. Group sizes: n = 6–14.

HE staining indicated signs of pancreatic fibrosis in the patchy atrophic areas in male *Cel-HYB1* mice but not in females. Fibrosis development in males was confirmed by Masson's trichrome staining of pancreatic sections (Fig. 4A). Immunohistochemistry for the marker F4/80 revealed that the inflammatory infiltrates consisted of macrophages (Fig. 4B). Leukocyte (CD45) and B-lymphocyte (CD45R) markers were positive in sections both from 6- and 12-month-old *Cel-HYB1* mice (Supplementary Fig. 6).

Next, we evaluated serum amylase, a pancreatitis marker. Amylase activity was measured in blood samples from male control and *Cel-HYB1* mice at ages 1, 3, 6 and 12 months. We found a significant increase in activity at 6 and 12 months in heterozygous *Cel-HYB1* mice, and the same trend was observed for the homozygous animals (Fig. 4C). Moreover, we evaluated intrapancreatic trypsin activity in pancreas homogenates from mice aged 6 months. Notably, there was no increase in trypsin activity in pancreata from *Cel*^*+/HYB1*^ and *Cel*^*HYB1/HYB1*^ animals compared to controls (Fig. 4D). This observation suggested that intrapancreatic trypsinogen activation does not play a central role in the disease mechanism.

In summary, the findings in *Cel-HYB1* mice were fully consistent with spontaneous development of CP. Moreover, pathological changes were observed in both hetero- and homozygous animals and became more severe as the mice aged.

### 3.5. Glucose homeostasis in Cel-HYB1 mice

**Fig. 5.**
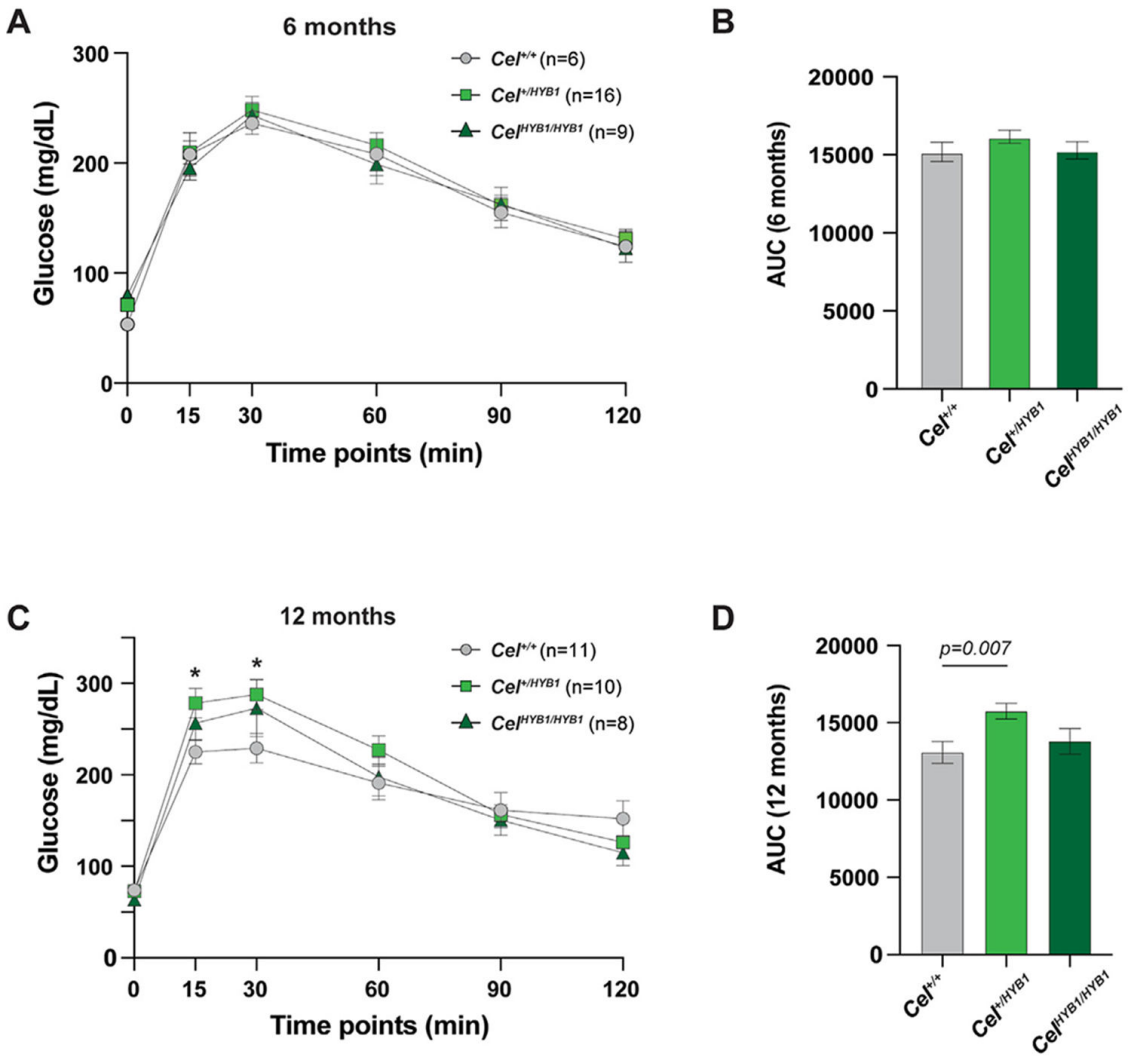
Glucose homeostasis in *Cel-HYB1* mice. (A) Intraperitoneal glucose tolerance test of male *Cel-HYB1* mice at age 6 months. (B) Comparison of area under the curve (AUC) for the mice in A. (C) Same experiment for male mice at age 12 months (D) Comparison of AUC for the mice in C. Results are shown as mean ± SEM. *p < 0.05.

As some *CEL* mutations are known to cause diabetes [12,13,25], we monitored endocrine function in the *Cel-HYB1* mice by measuring random blood glucose every other week. At no time point was there any indication of diabetes development (data not shown). To further analyze pancreatic endocrine function, we performed intraperitoneal glucose tolerance testing (IPGTT). At age 6 months, we observed no signs of altered glucose homeostasis in the *Cel-HYB1* mice (Fig. 5A and B and Supplementary Figs. 5B and C). At 12 months, however, heterozygous male *Cel-HYB1* mice had significantly elevated glucose levels after 15 min (*p* = 0.018) and after 30 min (*p* = 0.020) compared to their littermate controls (Fig. 5C). In addition, the area under the curve (AUC) was significantly increased for 12-month heterozygous males compared to controls (Fig. 5D). The IPGGT experiment indicated a similar effect in homozygous mice but differences between groups were not significant (Fig. 5C and D).

### 3.6. Cel protein aggregation and ER stress in Cel-HYB1 mice

**Fig. 6.**
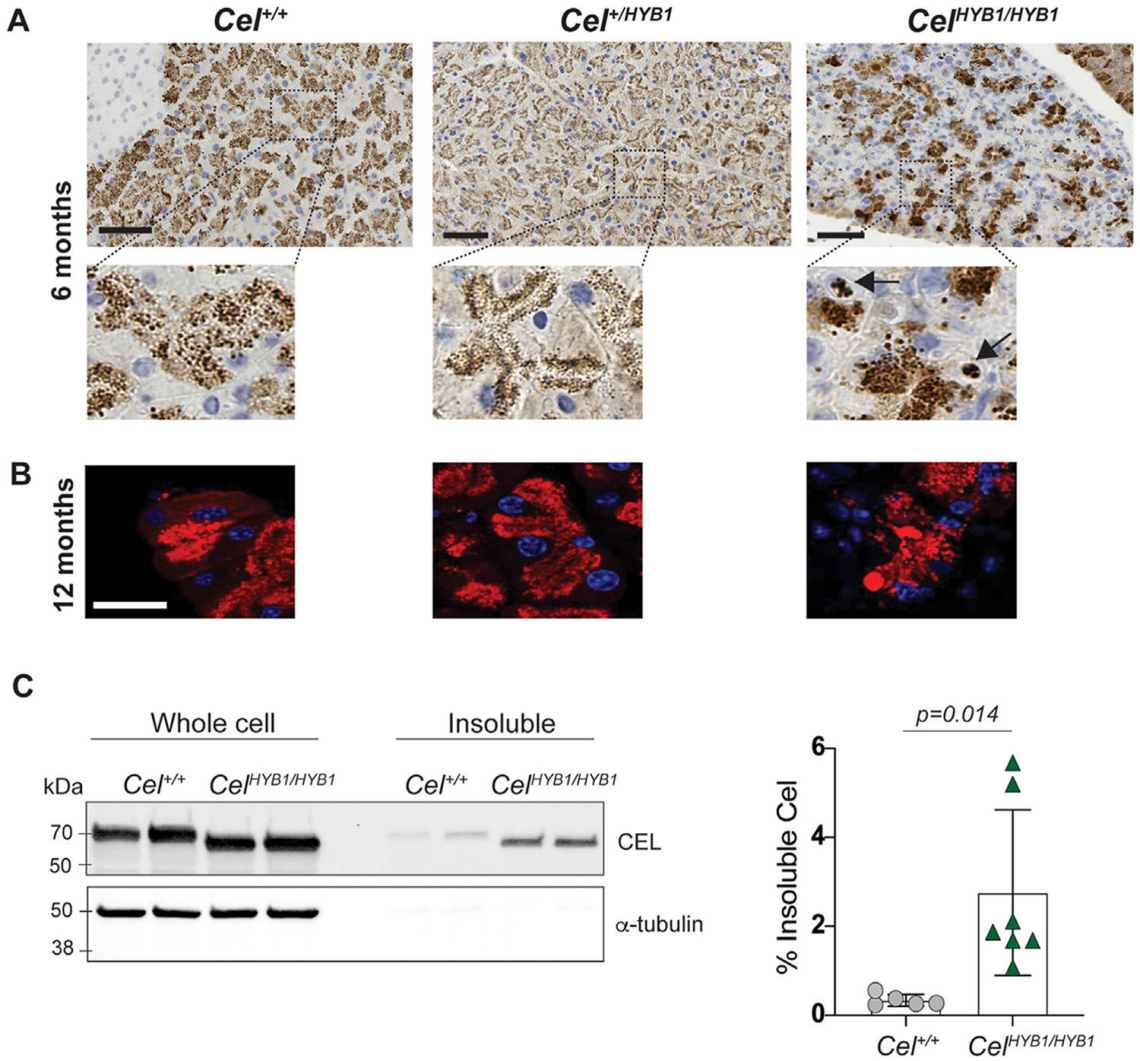
Distribution of CEL protein in the pancreas of *Cel-HYB1* mice. Chromogenic (A) and fluorescent (B) immunostaining with anti-CEL antibody. Representative pancreatic sections are shown from male control (*Cel*^*+/+*^), heterozygous (*Cel*^*+/HYB1*^) and homozygous (*Cel*^*HYB1/HYB1*^) animals. Nuclei are stained blue with DAPI in (B). Scale bars are 50 μm. (C) Expression of mCEL and mCEL-HYB1 proteins in *Cel*^*+/+*^ and *Cel*^*HYB1/HYB1*^ male mice (6 months old), respectively. Whole-cell lysates and insoluble pellet fractions were isolated from the pancreas, and blots were immunostained by an antibody towards the CEL globular domain. α-tubulin was visualized as loading control. Quantification of band intensities after normalizing to the intensity of the mCEL band is presented in the rightmost panel. Error bars are ±SD.

We have previously shown that the human CEL-HYB1 variant, but not normal CEL protein, aggregates in cellular lysates when expressed in HEK293 cells [16,22]. The generation of the *Cel-HYB1* mouse allowed us to follow up on this finding *in vivo*. First, CEL was visualized in pancreatic sections by immunohistochemistry (Fig. 6A) and immunofluorescence (Fig. 6B). In control mice, the protein localized to zymogen granules that were distributed as expected. In heterozygous *Cel*^*+/HYB1*^ mice, the positive zymogen granules showed a more apical distribution with the strongest granule staining lining the membrane. In the atrophied pancreatic lobules of homozygous mice, we observed loss of CEL expression in some cells whereas other cells exhibited distinct, positive puncta that were larger and more irregular than zymogen granules (Fig. 6A and B). Moreover, analysis by immunoblotting showed increased levels of CEL in the insoluble cell lysate fraction of pancreas homogenates obtained from *Cel*^*HYB1/HYB1*^ animals (Fig. 6C). Taken together, our observations suggest that mCEL-HYB1 forms intracellular protein aggregates in the acinar cells of the mouse pancreas.

**Fig. 7.**
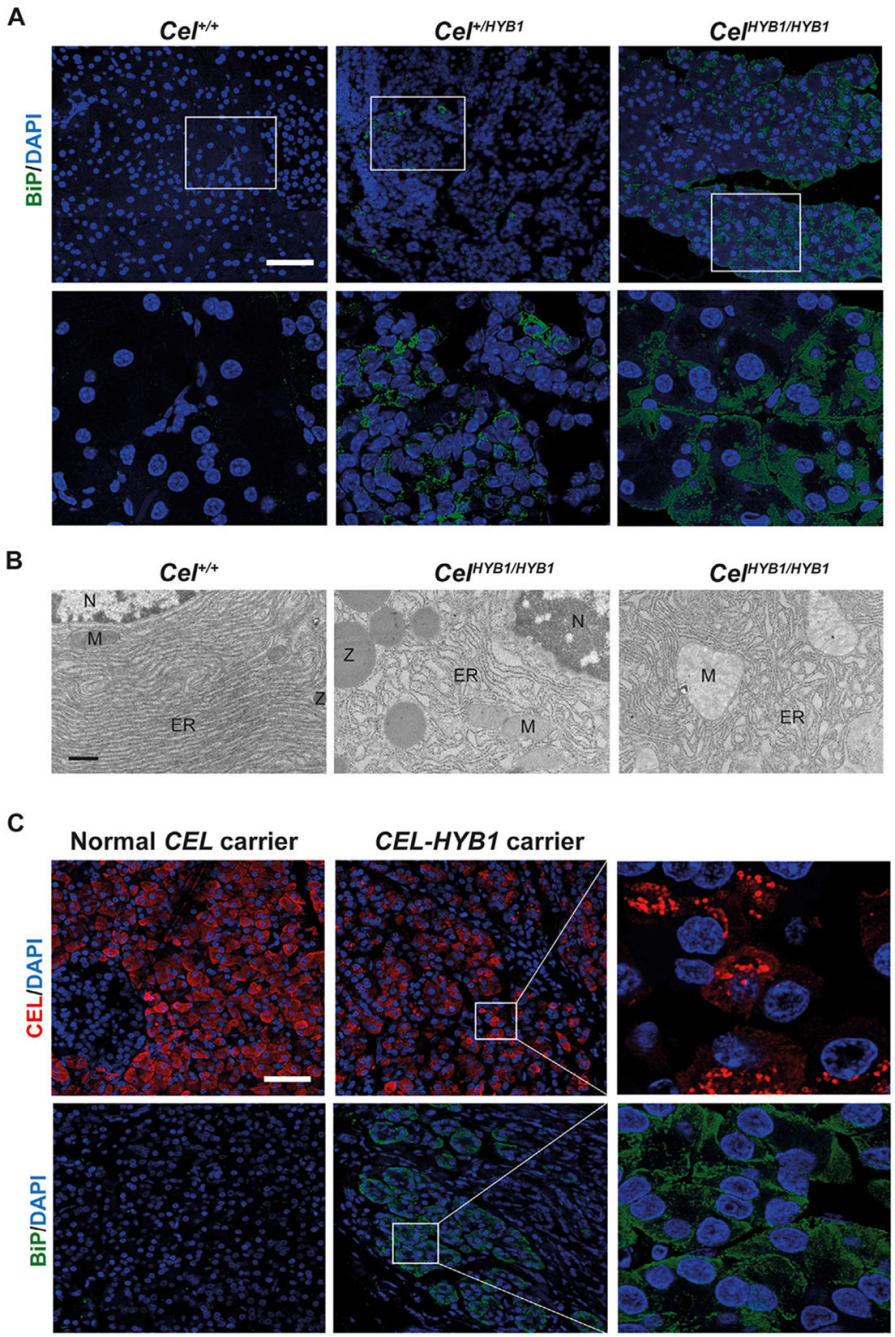
Induction of the ER stress marker BiP in pancreatic tissue expressing mCEL-HYB1. (A) Immunofluorescent staining for BiP protein (green). Representative pancreatic sections are shown from male controls (*Cel*^*+/+*^), heterozygous (*Cel*^*+/HYB1*^) and homozygous (*Cel*^*HYB1/HYB1*^) animals at age 6 months. The lower panels are higher magnifications of the rectangles in the upper panel. Scale bar is 50 μm. (B) Electron micrographs of acinar cells from a *Cel*^*HYB1/HYB1*^ female mouse aged 6 months. The right panel shows dilated ER and swollen mitochondria, whereas the middle panel shows dilated ER and normal mitochondria. A normal acinar cell from a Cel^*+/+*^ animal is presented in the left panel. Scale bar is 600 nm. (C) Immunostaining of CEL (red) and BiP (green) on sections from two patients with pancreatic ductal adenocarcinoma that either carried the two normal *CEL* alleles or was heterozygous for *CEL-HYB1* allele. Scale bar is 50 μm. Nuclei are stained blue with DAPI in (A) and (C).

Our previous studies of human CEL-HYB1 expressed in HEK293 cells had also indicated that this protein variant could induce ER stress [16,22]. Pancreas sections from the mouse model were therefore analyzed for the ER stress marker BiP, also known as glucose-regulated protein 78 (GRP78), by immunofluorescent staining, followed by confocal imaging. Increased BiP signals were detected both in hetero- and homozygous *Cel-HYB1* mice when compared with control animals (Fig. 7A). In contrast, when measuring pancreatic mRNA expression of *Hspa5* (encoding BiP) and the transcription factor *Ddit3* (encoding CHOP) in mice at 6 months of age, no difference was detected between homozygous *Cel-HYB1* mice and controls (Supplementary Fig. 7). However, further analysis by transmission electron microscopy of pancreatic tissue demonstrated dilated ER as well as swollen, damaged mitochondria in acinar cells of homozygous *Cel-HYB1* mice (Fig. 7B). Finally, tissue sections were available from a patient with pancreatic ductal adenocarcinoma who was a carrier of the *CEL-HYB1* allele and underwent surgery. In areas of atrophic pancreatic parenchyma outside the tumor, similar observations were made as for the *Cel-HYB1* mouse, namely punctate and variegated CEL positivity and upregulated BiP protein (Fig. 7C). This was not seen in pancreatic sections from similar patients who had undergone pancreatic surgery and were not *CEL-HYB1* carriers.

## 4. Discussion

Genetic association studies suggest that human carriers of the *CEL-HYB1* allele have increased risk for CP [15,17], but direct evidence linking *CEL-HYB1* to the pathophysiology of CP has been lacking. To test whether expression of *CEL-HYB1* causes CP, we used homologous recombination to replace the mouse *Cel* VNTR in exon 11 with the corresponding region of the human *CEL-HYB1* allele. The new model exhibited pancreatic expression of the humanized mCEL-HYB1 protein, demonstrated by western blotting and an antibody specific for the CEL-HYB1 tail sequence (Fig. 1D). Heterozygous and homozygous *Cel-HYB1* mice of both sexes spontaneously developed CP as evidenced by inflammation, acinar cell loss and fatty change (Fig. 3; Fig. 4A and B and Supplementary Fig. 5A). Our findings therefore provide direct evidence that expression of the *CEL-HYB1* VNTR causes CP. Notably, fibrosis seemed less pronounced in our model than in other animal CP models [26-28] and in human CP [29].

Fatty replacement was a characteristic feature of the affected pancreas in the *Cel-HYB1* mice. Pancreatic lipomatosis is frequently seen in human CP and can be prominent in pancreatitis associated with risk variants of the *PRSS1* and *CEL* genes, including *CEL-HYB1* [16,30,31]. The mechanism of fatty replacement in CP remains poorly defined. Whether the process results from adipocyte infiltration from peripancreatic fat or another mechanism like acinar-to-adipocyte transdifferentiation, has not been established [32]. With either mechanism, factors secreted from the injured pancreas could be positively associated with intrapancreatic fat deposition [33].

Compared to controls, our *Cel-HYB1* mice showed differences in the mass of fat depots also outside the pancreas (Fig. 2C, Supplementary Fig. 3C). These changes, however, differed between male and female mice. Homozygous *Cel-HYB1* male mice had significant increases in both white and brown fat depots, whereas female mice had decreased white fat mass and preserved brown fat mass. In addition, male and female mice exhibited different patterns of change in bodyweight and relative pancreatic weight. We did not explore the mechanisms behind the observed sex-related differences further. Variations between males and females with regard to the phenotype of pancreatitis have been described in the literature, however, the underlying causes remains unclear [34].

On the other hand, we investigated how expression of mCEL-HYB1 may cause acinar cell injury (Fig. 6; Fig. 7). Currently, two mechanisms dominate theories for the pathogenesis of CP [3,5]. The oldest, the trypsin-dependent model, posits that premature activation of trypsinogen to trypsin triggers a cascade of events ending in acinar cell death. More recently, the protein-misfolding model has gained attention. The first mouse model of spontaneous CP associated with protein misfolding was the *CPA1 N256K* knock-in mouse [28]. *CPA1* encodes the digestive enzyme carboxypeptidase A1, and in the *CPA1* mutant mouse, classical histological signs of CP were observed including acinar cell atrophy, inflammation, fibrosis and acinar-ductal metaplasia. Moreover, misfolding of the CPA1 protein resulted in diminished CPA1 secretion, intracellular CPA1 accumulation and elevated ER stress in the mouse acinar cells [28]. Interestingly, high trypsin activity was observed in the pancreata of these mice which may suggest that inappropriate trypsin activation is part of the disease mechanism. There was no difference in intrapancreatic trypsin activity in our *Cel-HYB1* and control mice. However, we measured trypsin in frozen tissue specimens and therefore cannot rule out that freezing and thawing may have affected the result.

Instead, we found evidence of CEL-HYB1 protein misfolding. Our published results of cultured cells expressing the human CEL-HYB1 protein provided strong support that CEL-HYB1 misfolds and activates ER stress [22]. In the current study, immunohistochemistry performed on mouse pancreatic sections with an antibody specific for CEL-HYB1 showed mCEL-HYB1-positive puncta in homozygous animals. The puncta were distinct from zymogen granules in appearance, which suggested they were aggregates of mCEL-HYB1 protein (Fig. 6A and B). We also separated pancreatic extracts obtained from homozygous and wildtype animals into detergent-soluble and detergent-insoluble fractions. This procedure detected significantly increased levels of mCEL-HYB1 compared with normal mCEL in the detergent-insoluble fraction (Fig. 6C). Both observations are consistent with aggregation of misfolded mCEL-HYB1.

Moreover, immunostaining showed that expression of the ER stress marker BiP was increased in acinar cells of heterozygous and homozygous *Cel-HYB1* mice at age 6 months (Fig. 7A). This result was not confirmed at the RNA level (Supplementary Fig. 7). The reason is probably due to the patchiness of tissue changes in the *Cel-HYB1* mice and that upregulation of ER stress markers first and foremost happens in restricted atrophic areas at this age, while the mRNA was extracted from bulk pancreatic tissue. To support this argument, we observed dilated ER in acinar cells of *Cel-HYB1* animals by transmission electron microscopy, a direct measure of ER stress (Fig. 7B). In addition, swollen, damaged mitochondria consistent with outer membrane permeabilization were frequently present, suggesting that the ER stress might trigger activation of the maladaptive, intrinsic apoptotic pathway [35].

Importantly, we extended our studies to the human pancreas by immunostaining pancreatic sections obtained from a *CEL-HYB1* carrier (Fig. 7C). Staining for CEL showed irregular puncta that were larger than zymogen granules and similar to the changes seen in *Cel-HYB1* mice, suggesting aggregation of human CEL-HYB1 protein. In addition, the level of BiP in cells expressing CEL-HYB1 was increased over the level detected in control pancreata expressing normal CEL protein. Taken together, our findings support the conclusion that CEL-HYB1-associated CP is a protein-misfolding disease in humans. The role of ER stress in misfolding-induced CP, however, can be debated. In a recent study, the development of CP in *CPA1 N256K* mutant mice was found to be unaffected when the ER stress gene *Ddit/Chop* was globally deleted [36]. Thus, the mild ER stress found in both *CPA1 N256K* and *CEL-HYB1* mice may not be pathogenic – protein misfolding could cause pancreatitis via other mechanisms.

While this manuscript was in preparation, another group independently published the characterization of a mouse model of *CEL-HYB1* expression [37]. Their model was created by a CRISPR/Cas9 approach, by inserting the cDNA encoding human *CEL-HYB1* into exon 2 of mouse *Cel*. The animals therefore express the human CEL-HYB1 protein with a mouse signal peptide. Moreover, to enable efficient detection of the CEL protein, a 3xFlag tag sequence was added after the *CEL-HYB1* VNTR [37]. Even so, similar pancreatic pathology is observed between that model and the mice of our study, with patchy injury characterized by fibrosis and inflammation. The main histological difference was greater extent of fatty change in our model, similar to findings in patients with pathogenic *CEL* mutations [16,31]. Like our report, Mao et al. observed no increase in intrapancreatic trypsin activity and instead presented evidence for protein misfolding as the underlying pathophysiology of *CEL-HYB1*-associated CP [37]. In addition to dilated ER, which both studies demonstrated, we indirectly show mitochondrial outer membrane permeabilization consistent with activation of the intrinsic apoptosis pathway [38]. Another new contribution from the present study is the immunostaining of pancreatic sections from a *CEL-HYB1* carrier, which directly supports misfolding of the pathogenic CEL protein in human disease.

Notably, none of the two *CEL-HYB1* mouse models contain the rare SNPs of CEL exon 10 (rs77696629 T>C, p.Ile488Thr) and exon 11 (rs750991274, p. Thr548Ile), which previously have shown to be present in the majority of human carriers of the *CEL-HYB1* allele [22]. This suggests that it is the VNTR sequence of *CEL-HYB1* that is the main driver of pathogenicity. Still, it cannot be excluded that the threonine and isoleucine in position 488 and 548, respectively, have some specific effects in the context of human CP as these variant, when tested in a cellular model system, affected the properties of the CEL protein significantly [22]. An interesting follow-up study would therefore be to introduce these SNPs into our *Cel-HYB1* mouse model, to see if they will increase proteotoxicity and worsen the phenotype.

Finally, in contrast to Mao et al. [37], we characterized endocrine pancreatic function. There were no signs of elevated random or fasting blood sugar levels in our model throughout the course of exocrine pancreatic disease. However, we noted impaired glucose tolerance in male mice aged 12 months. Glucose levels were significantly elevated 15 and 30 min after glucose injection, although they normalized after 60 min (Fig. 5C and D). Diabetes is a common complication of CP in humans [39], whereas CP mouse models tend to be normoglycemic when investigated [26]. In humans, diabetes seems to be an inevitable consequence of the single-base deletions in the *CEL* VNTR that cause the hereditary disease MODY8 [12,13]. Due to too few animals, we were not able to analyze the endocrine function of *Cel-HYB1* female mice at 12 months, which is a limitation of this study. Thus, it remains to be seen whether the impaired glucose tolerance observed in *Cel-HYB1* males at age 12 months can be confirmed in females, and if the mice eventually will develop diabetes.

In summary, we present a new preclinical model of *CEL-HYB1*-associated CP. We also provide evidence that the disease mechanism is related to proteotoxic protein misfolding rather to activation of trypsin-dependent pathways, both in our mouse model and in the human pancreas. Thus, our mouse model of *CEL-HYB1*-associated CP spontaneously recapitulates the pathophysiology in humans and offers an important tool for future studies of cellular pathways mediating pancreatic inflammatory disease, for interrogating effects on glucose homeostasis, and for testing potential therapies.

## Supplementary Material

Supp Table 1

Supp Fig 1

Supp Fig 2

Supp Fig 3

Supp Fig 4

Supp Fig 5

Supp Fig 6

Supp Fig 7
