# Supplementary material for: The genetic risk factor CEL-HYB1 causes proteotoxicity and chronic pancreatitis in mice
Source: Pancreatology. Author manuscript; Available in PMC 2024 Jun 7. (PMC11157984; doi:10.1016/j.pan.2022.11.003)
Supplement: Supp Table 1 [file NIHMS1996046-supplement-Supp_Table_1.pdf]

Suppl. Table 1. Histology scores of pancreatic sections from *Cel-HYB1* mice

MALES, 6 months

Scores	+/+ n = 4	+/*HYB1* n = 11	*HYB1/HYB1* n = 11
**Inflammation**			
0	4	8	5
1		3	4
2			2
**Fatty replacement**			
0	4	11	8
1			3
2			
**Acinar atrophy**			
0	4	11	8
1			2
2			1

MALES, 12 months

Scores	+/+ n = 5	+/*HYB1* n = 10	*HYB1/HYB1* n = 9
**Inflammation**			
0	3	1	
1	2	3	6
2		6	3
**Fatty replacement**			
0	4	2	1
1	1	5	6
2		3	2
**Acinar atrophy**			
0	5	3	1
1		2	6
2		5	2

FEMALES, 6 months

Scores	+/+ n = 7	+/*HYB1* n = 14	*HYB1/HYB1* n = 9
**Inflammation**			
0	7	4	3
1		6	5
2		4	1
**Fatty replacement**			
0	6	13	8
1	1		1
2		1	
**Acinar atrophy**			
0	7	12	8
1		1	1
2		1	

FEMALES, 12 months

Scores	+/+ n = 1	+/*HYB1* n = 2	*HYB1/HYB1* n = 4
**Inflammation**			
0			
1	1	2	1
2			3
**Fatty replacement**			
0	1	2	2
1			2
2			
**Acinar atrophy**			
0	1	2	3
1			
2			1

One representative pancreatic section from each animal was scored by an expert pancreas pathologist without access to genotype, age or sex of the mice. The scores were: **0**, normal morphology; **1**, mild to moderate changes; **2**, extensive changes.
