# Supplementary material for: The genetic risk factor CEL-HYB1 causes proteotoxicity and chronic pancreatitis in mice
Source: Pancreatology. Author manuscript; Available in PMC 2024 Jun 7. (PMC11157984; doi:10.1016/j.pan.2022.11.003)
Supplement: Supp Fig 1 [file NIHMS1996046-supplement-Supp_Fig_1.pdf]

Downstream LoxP site (34 bp)

ATAACTTCGTATAATGTATGCTATACGAAGTTATactagtgatatcactacatggtgctcctgaaatcattc

cgcagagctgtccaggagcacgggcctccagtctctttgcaggcccctgctcagtctcttctcactctgcag

D P N M G N S P V P T H W Y P Y T L E N G N Y L

GGACCCCAACATGGGCAACTCACCTGTGCCCACACACTGGTACCCTTATACCTTGGAGAATGGCAACTACCT

D I T K T I T S A S M K E H L R E K F L K F W A

GGACATCACTAAGACGATAACCAGCGCTTCCATGAAGGAGCACCTGAGAGAAAAGTTCCTGAAGTTCTGGGC

V T F E V L P T V T D Q E A S S M P S T G D S E

TGTGACATTCGAGGTGCTGCCCACAGTGACTGACCAGGAGGCCAGTTCCATGCCCTCCACAGGGGACTCTGA

A T P V S P D R Q L R V C P R P C N G **X**

GGCCACTCCCGTCTCCCCCGACAGGCAACTCCGAGTCTGCCCCCGTCCCTGCAACGGGtagctcgagagtct

tataagctggggctggagagatgggtcaggaattaagatcactcactgttcttctaaaggtcctgtgttcaa

ttcccagcacctacatggctgctcacaactgtctgactccatctactggtgtgcagatgtacatgcagacta

agtacccatatacataaaatacatacataaataaagtcatataagccttggtcccagggttctgtttcaata

aagctctccttcaataaagcctcagccctcactatctgataattt
