# Supplementary figures and images for: The genetic risk factor CEL-HYB1 causes proteotoxicity and chronic pancreatitis in mice
Source: Pancreatology. Author manuscript; Available in PMC 2024 Jun 7. (PMC11157984; doi:10.1016/j.pan.2022.11.003)

**A**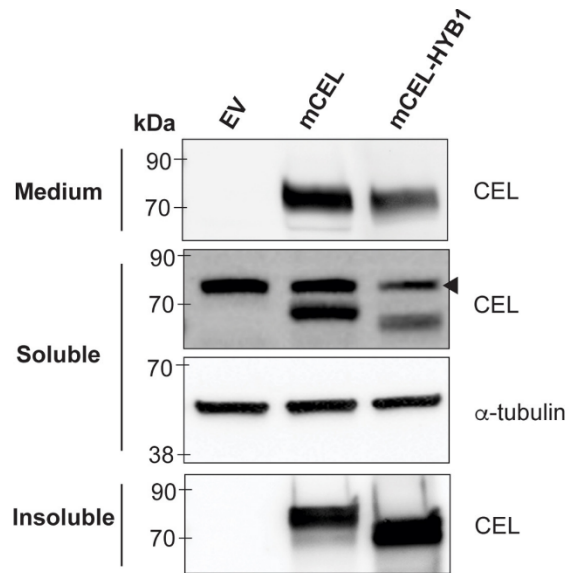**B**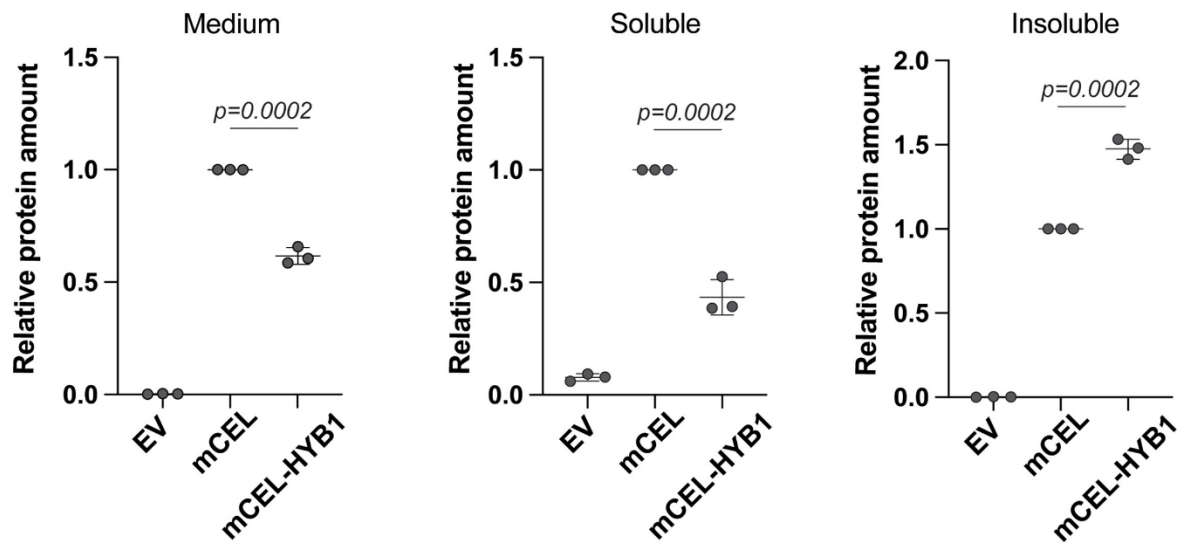**C**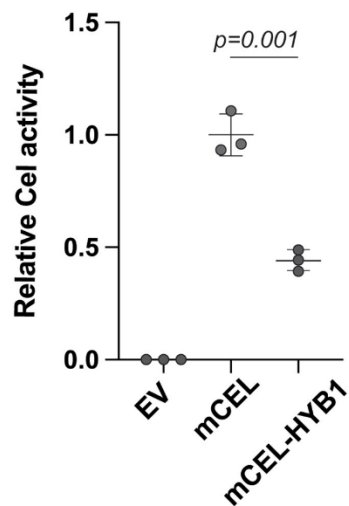

Supplement: Supp Fig 2 [file NIHMS1996046-supplement-Supp_Fig_2.pdf]

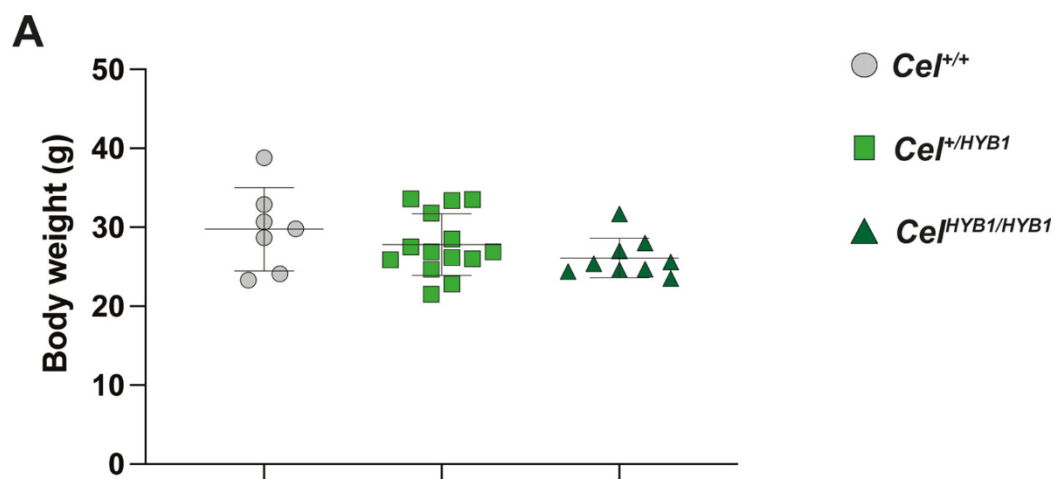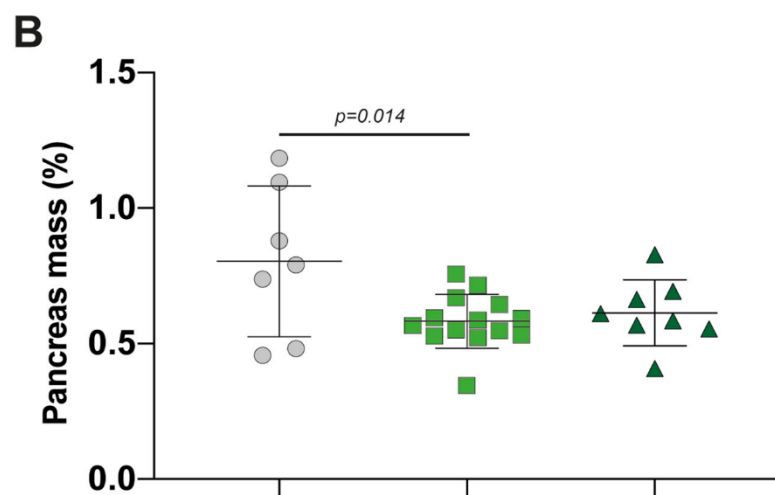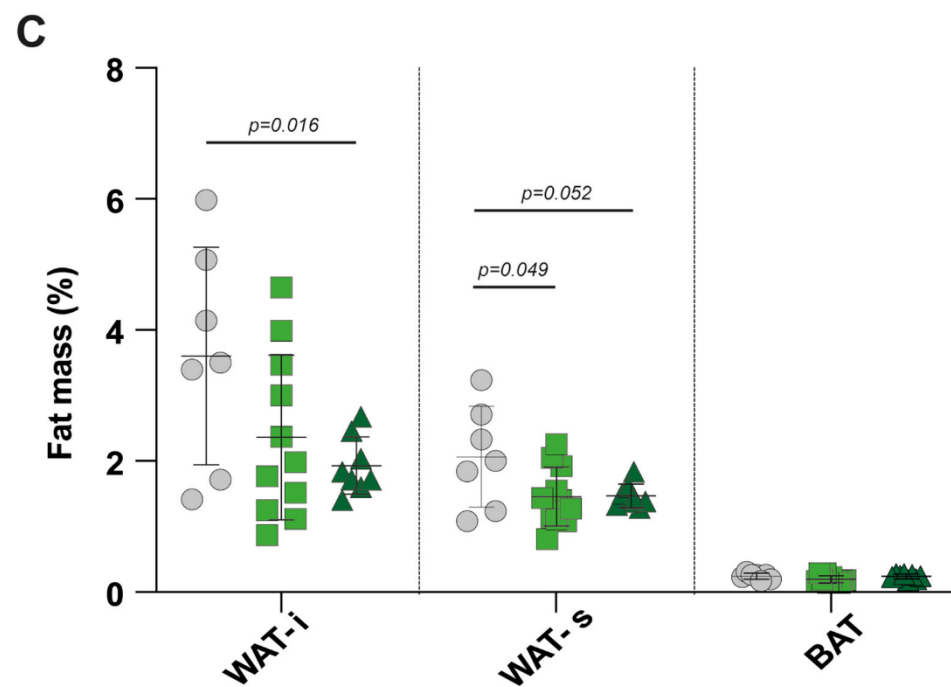

Supplement: Supp Fig 3 [file NIHMS1996046-supplement-Supp_Fig_3.pdf]

**A*****Cel*<sup>+/HYB1</sup>**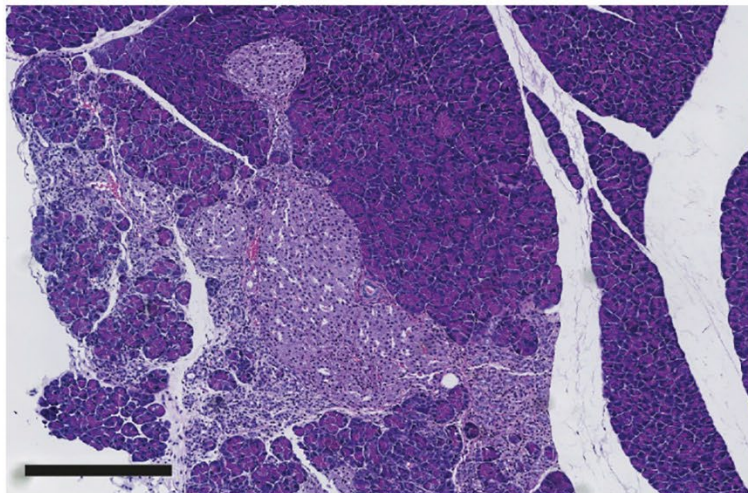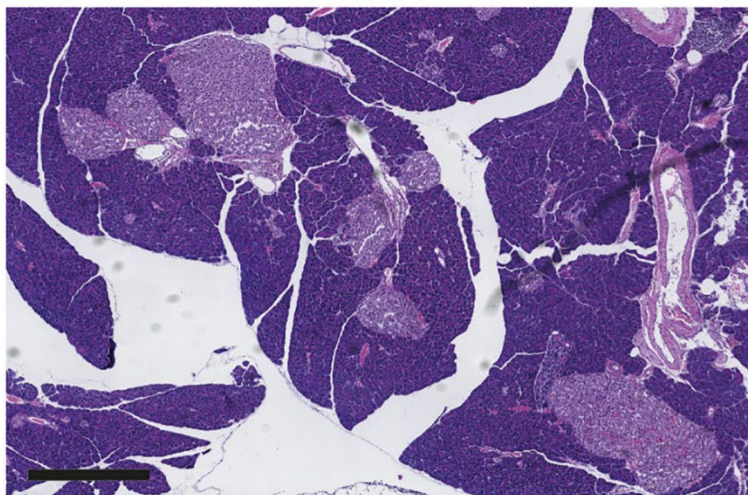**B*****Cel*<sup>HYB1/HYB1</sup>**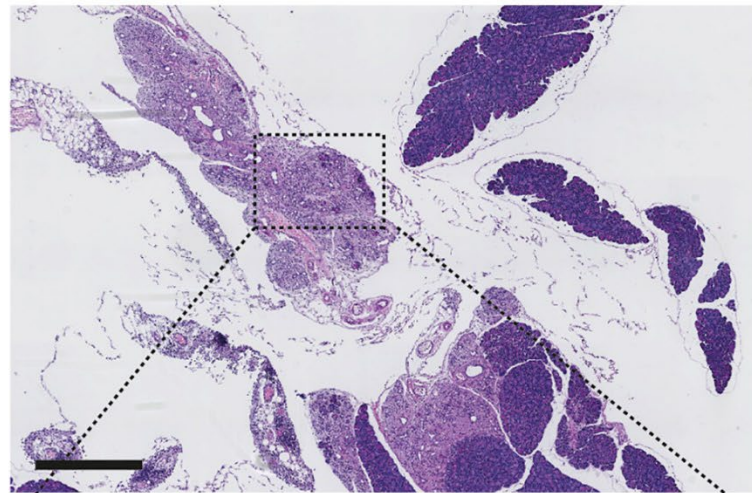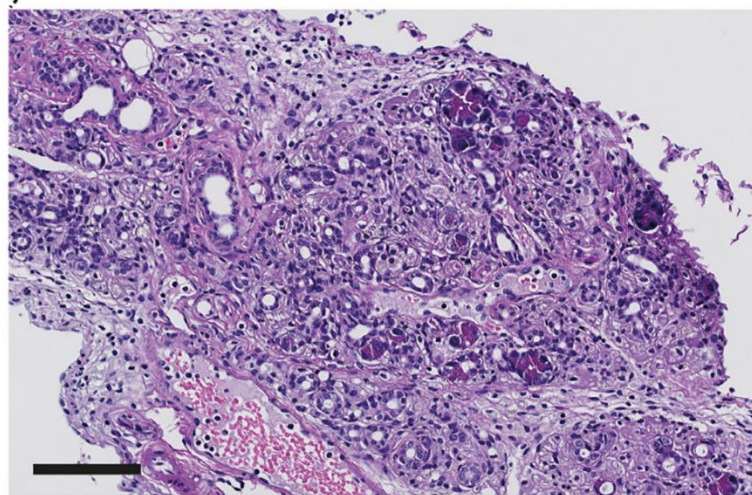

Supplement: Supp Fig 4 [file NIHMS1996046-supplement-Supp_Fig_4.pdf]

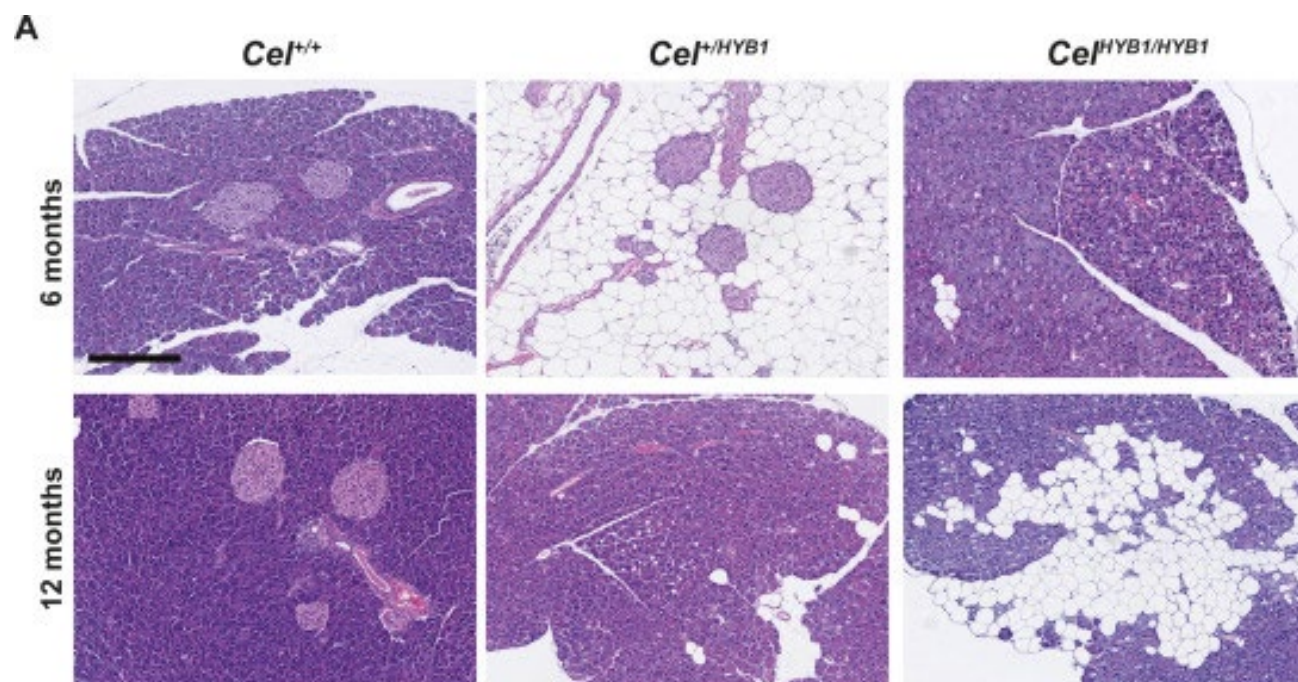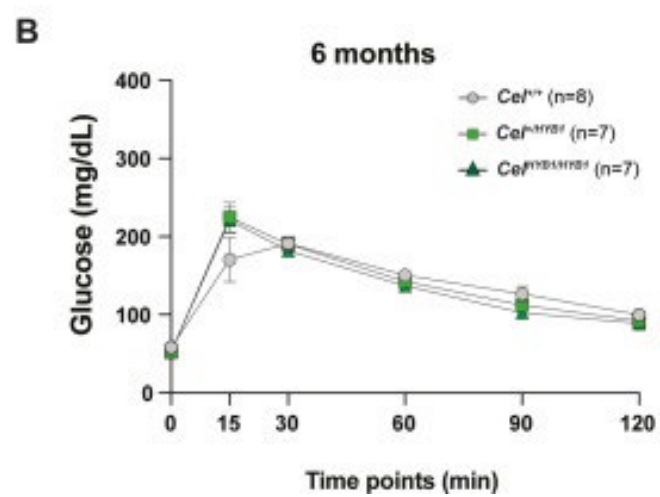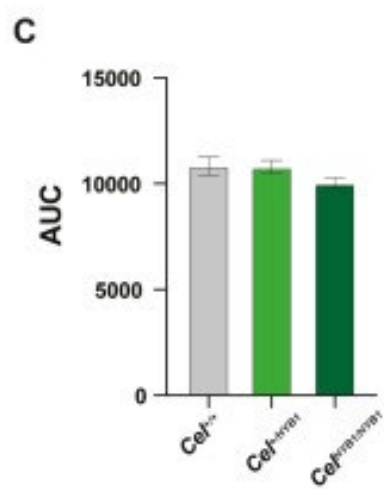

Supplement: Supp Fig 5 [file NIHMS1996046-supplement-Supp_Fig_5.pdf]

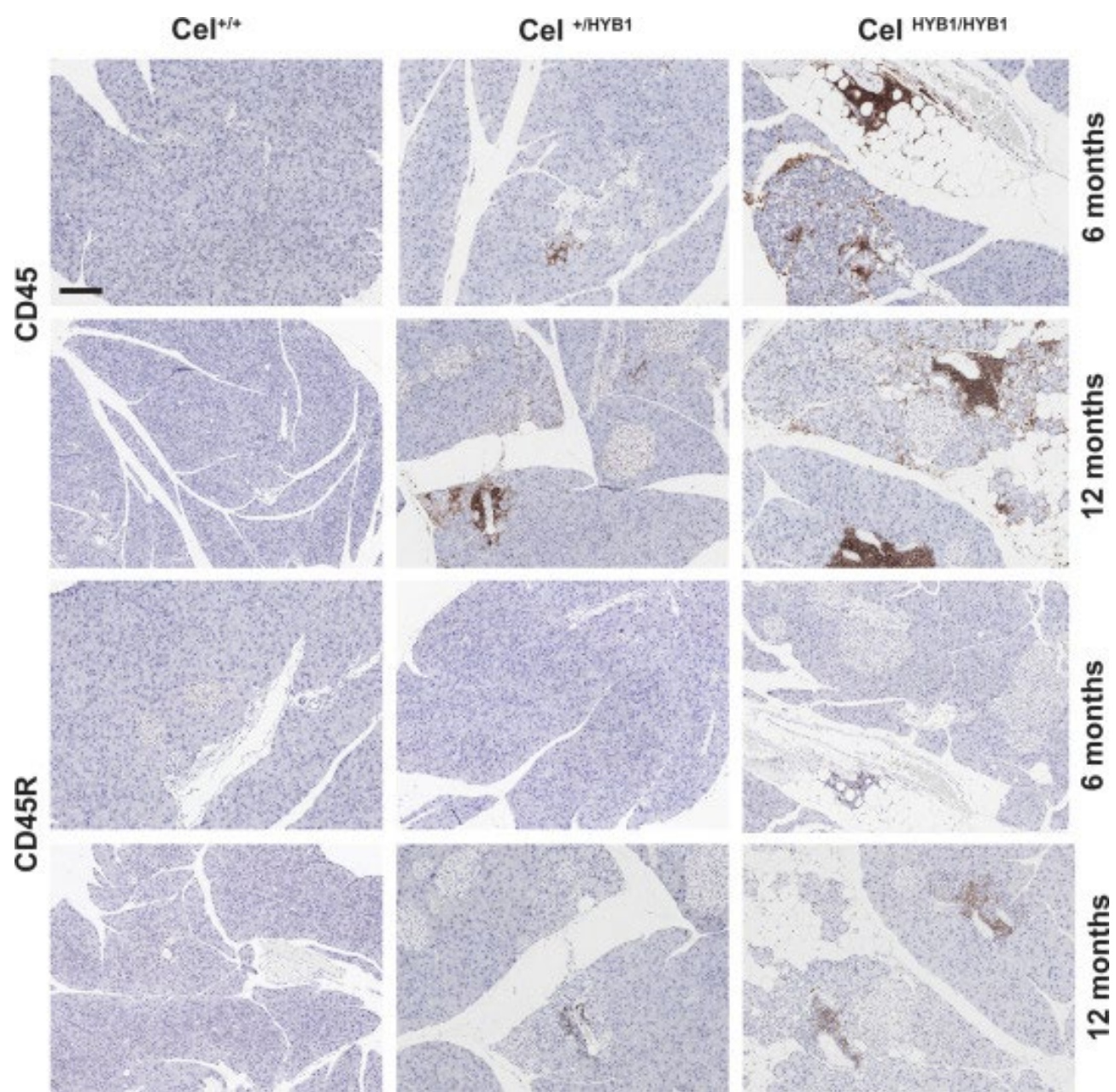

Supplement: Supp Fig 6 [file NIHMS1996046-supplement-Supp_Fig_6.pdf]

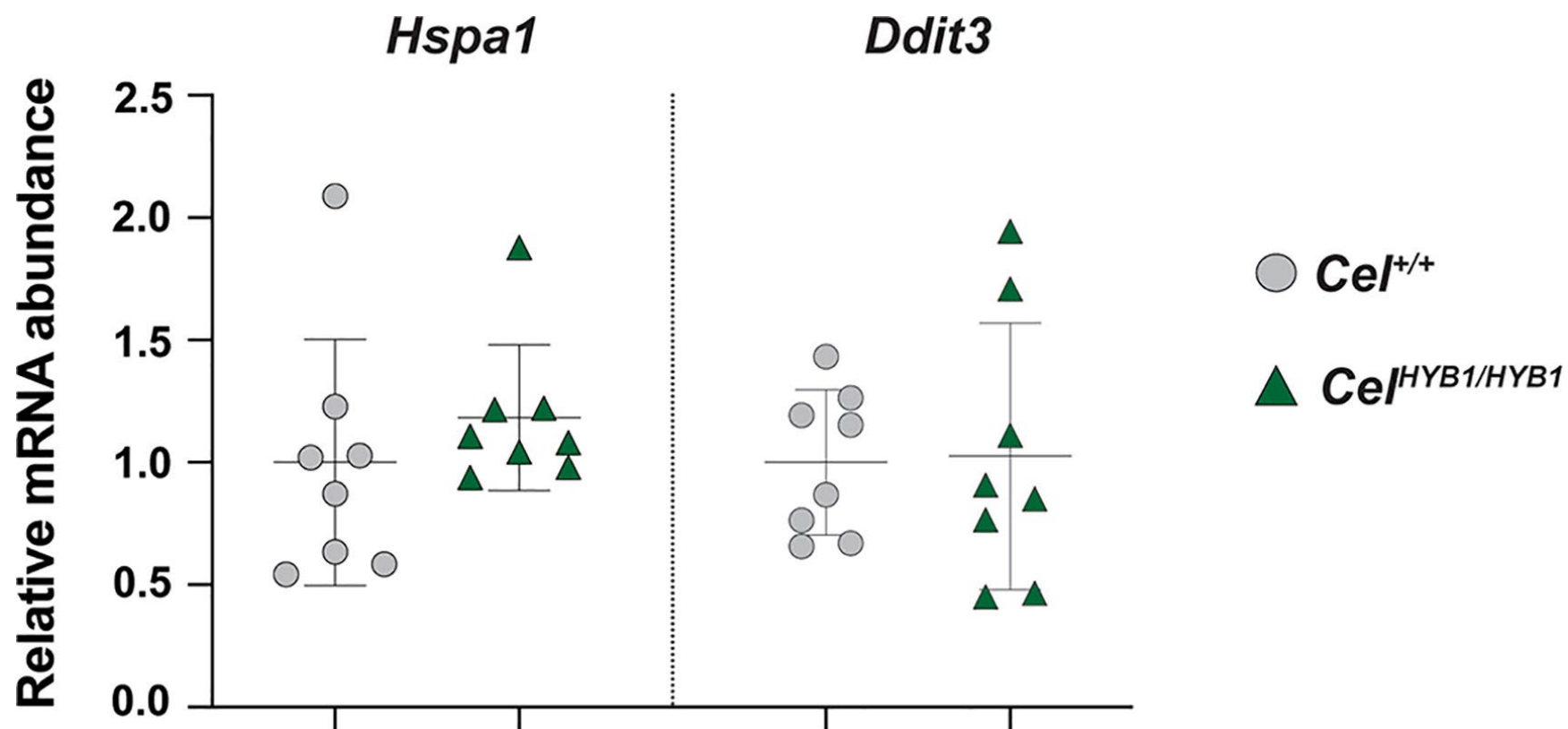

Supplement: Supp Fig 7 [file NIHMS1996046-supplement-Supp_Fig_7.pdf]
